# The Reconstruction of Sesame Protein-Derived Amyloid Fibrils Alleviates the Gastric Digestion Instability of β-Carotene Nanoparticles

**DOI:** 10.3390/nano15231829

**Published:** 2025-12-03

**Authors:** Liang Zhang, Puxuan Zhang, Haocheng Tong, Yue Zhao, Tengfei Yu, Guanchen Liu, Donghong Liu

**Affiliations:** 1 College of Biosystems Engineering and Food Science, Zhejiang University, Hangzhou 310058, China; 2 Innovation Center of Yangtze River Delta, Zhejiang University, Jiaxing 314100, China; 3 Technology Research Institute, CR Snow Breweries, Beijing 100010, China

**Keywords:** amyloid fibril, reconstruction, gastric digestion, β-carotene nanoparticles

## Abstract

In this study, the structural changes and reconstruction mechanism of sesame protein-derived amyloid fibrils under varied digestive parameters (pepsin concentration, digestive pH and ionic strength) during gastric digestion were investigated, and the effect of fibril reconstruction on the gastric digestion stability of β-carotene nanoparticles was also explored. The results demonstrated that amyloid fibrils underwent a three-stage dynamic process of enzymatic hydrolysis, regeneration and degradation during gastric digestion. The pepsin concentration of 2 mg/mL was found to promote the balance between fibril hydrolysis and regeneration. The fibrils displayed a pronounced regenerative capacity at pH values of 1.5 and 2.5, whereas at pH 3.5, which was proximal to the isoelectric point of protein, aggregation and precipitation were observed. Furthermore, it was found that 10 mM NaCl exerted minimal influence on fibril stability, whereas the higher concentrations of salt ions were shown to obstruct regeneration and promote aggregation. Analyses through SDS-PAGE, GPC, and MALDI-TOF-MS revealed a gradual reduction in the molecular weight of the fibrils during gastric digestion, with certain fragments reaggregating to form new fibril structures. The fibril-based delivery system formed a stable protective structure for β-carotene nanoparticles, which not only prevented their aggregation but also facilitated their release in the small intestine.

## 1. Introduction

In the food sector, amyloid fibrils with nanoscale are a type of highly ordered β-sheet structure aggregates formed by the partial folding and self-assembly of proteins, and they have now become a frontier research area in food-related functional nanomaterials [1]. When animal or plant proteins undergo denaturation and hydrolysis under acidic and heating conditions, polypeptides (i.e., building units) are released, and thereafter amyloid fibrils are generated based on a large number of β-sheet chains through layer-by-layer stacking [2]. In recent years, many researchers have conducted in-depth studies on the structural characterization, potential applications and edible safety of amyloid fibrils [3,4,5], demonstrating that amyloid fibrils are safe functional components and possess higher structural stability and functionalization potential.

In recent years, the formation mechanism and release property of amyloid fibril-based delivery carriers have gradually emerged as frontier scientific issues in food colloid science [6,7]. Generally, the impact of compositional and environmental variations during the digestion of amyloid fibrils is regarded as a dynamic process, primarily regulated by pH, digestive enzymes, ions and the mucus layer [8]. Several studies have shown that due to the unique cross-β-sheet structure of amyloid fibrils, their sensitivity to proteases is significantly reduced, endowing them with stronger resistance to digestive enzymes compared to native proteins [9,10,11]. However, the digestion rate of amyloid fibrils from different protein sources varies during simulated gastric digestion, depending on factors such as protein origin (animal/plant), spatial structure and fibril morphology [12]. Our previous study investigated the gastrointestinal digestion characteristics of amyloid fibrils derived from four plant-based oilseed proteins (soybean, sesame, walnut, and rapeseed) [13], revealing that these fibrils could effectively resist the adverse effects of gastric acid and pepsin with a low gastric digestion rate (5–10%). Nevertheless, the underlying mechanism of gastric digestion resistance of these amyloid fibrils remains unclear.

As a representative of poorly soluble ingredients, β-carotene has significant physiological benefits and broad economic prospects [14,15]. However, due to its poor stability during gastric digestion, especially in the harsh digestive environment of the stomach, β-carotene is prone to aggregation, degradation and isomerization [16,17,18]. The defects above hinder the further release and transport of β-carotene to the targeted site in the small intestine. Therefore, resisting the gastric digestive barrier (i.e., gastric acid erosion, pepsin hydrolysis, gastric mucus interaction and changes in ionic strength) on the digestive stability of β-carotene is a key factor for improving its bioaccessibility in the small intestine [19,20]. Herein, amyloid fibrils derived from sesame protein are used to construct a transport carrier for β-carotene with a coronary structure. During gastric digestion, this carrier can resist the gastric digestion barrier of β-carotene nanoparticles through in situ recombination of fibrils, thereby providing better gastric digestion stability and small intestine sustained-release performance for β-carotene. The content of this research may provide a theoretical basis for elucidating the gastric digestion resistance of amyloid fibrils and digestive stabilization of β-carotene loaded carriers.

## 2. Materials and Methods

### 2.1. Materials

Sesame protein (purity 90.3%) was purchased from Xinyiran Bio-Tech Co., Ltd. (Xi’an, China). Food-grade β-carotene crystals (purity 96%) were purchased from Zhejiang medicine Co., Ltd. (Shaoxing, China). Protein marker (rainbow 180 broad spectrum, 11 kD~180 kD) was purchased from Solarbio Co., Ltd. (Beijing, China). Thioflavin T (ThT, Mw 318.86) was purchased from Aladdin Co., Ltd. (Shanghai, China). Digestive enzymes, including pepsin (porcine, 474 units/mg, P7000), bile salts (porcine, 48305) and pancreatin (porcine pancreas, P7545) were purchased from Merck Science Co., Ltd. (Shanghai, China). Other analytical-grade reagents were purchased from local reagent companies.

### 2.2. Preparation of Sesame Protein Amyloid Fibrils

Based on the previous study, sesame protein was selected for this experiment due to its high fibril conversion rate and digestion resistance among the four oilseed proteins [13]. Sesame protein amyloid fibrils were prepared using the acid-heating method. Briefly, sesame protein was dispersed in distilled water to obtain a 25 mg/mL protein solution. The solution pH was adjusted to 9.5 and stirred at 50 °C for 3 h. Then, the pH was adjusted to 2.0 by the preconfigured 1.0 M hydrochloric acid solution (HCl), and the solution was centrifuged at 6000 rpm for 20 min to remove insoluble substances. The supernatant was heated at pH 2.0, 250 rpm and 85 °C for 24 h to form mature amyloid fibrils. The fibril dispersion was then subjected to a dialysis procedure in pH 2.0 distilled water as follows. Firstly, the prepared fibril dispersion was transferred to a dialysis bag with a molecular weight cut-off of 5000 Da, and the bag was ensured to be tightly sealed to prevent leakage. Then, the dialysis bag was immersed in pH 2.0 distilled water at a stirring speed of 50 rpm, allowing the small molecular salt ions and unreacted peptide segments to slowly diffuse out of the bag under the gradient-driven concentration effect. The entire dialysis process was carried out at the ambient temperature (about 20 °C) and lasted for 24 h, and the dialysis solution was replaced once during this period. After the dialysis was completed, the fibril dispersion with a significant reduction in salts and unconverted peptide segments could be obtained. The resultant fibril dispersion was stored at 4 °C for further analysis.

### 2.3. Simulated Gastric Digestion of Sesame Protein Amyloid Fibrils

The gastric digestion characteristics of amyloid fibril dispersions were investigated using a dynamic simulated digestive system (GI20, Nutriscan, Condell Park, NSW, Australia) [10]. A 20 mL aliquot of 20 mg/mL sesame protein amyloid fibril dispersion was placed in a sample vial and maintained at 37 °C in a heating block. Then, 20 mL of simulated gastric fluid (adjusted to pH 2.0 with 1.0 M HCl, containing the specific concentrations of pepsin, NaCl and 2.0 mg/mL CaCl_2_) was pumped into the fibril dispersion at a rate of 2 mL/min using a peristaltic pump with simultaneous stirring at 200 rpm. During digestion, 1 mL aliquots were collected at 0, 2, 5, 10, 20, 30, 60, 90, and 120 min, and enzymes were inactivated by heating in a 95 °C water bath for 5 min for further analysis.

### 2.4. ThT Fluorescence Spectroscopy

ThT fluorescence intensity was used to reflect the formation process of amyloid fibril, following the method of Zhao et al. [21]. A ThT stock solution was prepared by dissolving 16 mg ThT in 20 mL of phosphate buffer (10 mM, pH 7.0) containing 150 mM NaCl. The stock solution was diluted 50-fold and filtered through a 0.22 μm membrane. Then, 4 mL of the ThT working solution was mixed with 40 μL of fibril dispersion (1 mg/mL). Fluorescence measurements were performed using a fluorescence spectrophotometer (FL6500, PerkinElmer, Shelton, CT, USA) with excitation and emission wavelengths of 440 nm and 482 nm, respectively. The fluorescence intensity of the ThT working solution was used as a control.

### 2.5. Transmission Electron Microscopy (TEM)

The microstructure of amyloid fibrils was observed using transmission electron microscopy (JEM-1400, Hitachi, Tokyo, Japan) [13]. The fibril dispersion was diluted to 0.1 mg/mL with distilled water (pH 2.0), and 20 μL of the diluted dispersion was transferred to a 300-mesh copper grid. After incubation at the ambient temperature and humidity of about 20 °C and 60%, respectively, for 15 min, residual moisture was removed using filter paper, and the sample was negatively stained with 3.0 wt% phosphotungstic acid for 10 min. After natural drying, the microstructure was observed and imaged at a scale of 100 nm.

### 2.6. Rheological Properties of Amyloid Fibril Digesta

The rheological properties of the amyloid fibril digesta were measured using a rheometer (MCR302, Anton Paar, Graz, Austria) equipped with a 40 mm parallel plate [22]. For steady-state flow tests, the viscosity of the sample was recorded within a shear rate range of 0.1–100 s^−1^. For oscillatory frequency sweeps, the linear viscoelastic region of the sample was preliminarily determined through dynamic strain scans. Then, within the linear viscoelastic region at a fixed strain of 1%, the storage modulus (G′) and loss modulus (G″) were recorded as functions of angular frequency (0.1–100 rad/s).

### 2.7. Sodium Dodecyl Sulfate Polyacrylamide Gel Electrophoresis (SDS-PAGE)

SDS-PAGE images of gastric digestion products of amyloid fibrils were obtained using a vertical electrophoresis tank (Mini-PROTEAN Tetra Cell, Bio-Rad, Hercules, CA, USA) [23]. Each sample was diluted to 10 mg/mL with distilled water and the pH was adjusted to 7.0. Then, 10 μL of 5× loading buffer (containing DTT) was mixed with 40 μL of the diluted dispersion and heated at 95 °C for 10 min for further analysis. Precast gels were prepared using a one-step PAGE gel rapid preparation kit (Yamei Biotech, Shanghai, China), with the stacking and separating gel concentrations of 4.5% and 6% (*v*/*v*), respectively. After gel solidification, protein markers (5.5 μL, 11–180 kDa) and processed fibril dispersions (10 μL) were loaded onto the gel. Electrophoresis was performed at 150 V for 70 min, followed by staining with Coomassie Brilliant Blue R250 for 30 min and destaining in distilled water overnight. Gel images were obtained using a gel imaging system (ChemiScope 6100, Shanghai, China).

### 2.8. Gel Permeation Chromatography (GPC)

GPC (PL-GPC220, Agilent, Santa Clara, CA, USA) was used to determine the molecular weight of amyloid fibril gastric digesta. Unlike SDS-PAGE, GPC avoids the use of strong denaturants to maintain the native conformation of proteins [24]. Samples were diluted with phosphate buffer to ensure complete dispersion without aggregation, and the fibril concentration was adjusted to 2 mg/mL. Impurities were removed using a 0.45 μm filter. After instrument preheating, the mobile phase was set as phosphate buffer with a flow rate of 1.0 mL/min and a column temperature of 25 °C. A calibration curve of retention time versus molecular weight was established using standard proteins of known molecular weights. A 100 μL sample was manually injected, and data acquisition started after baseline stabilization. Elution peaks were monitored at 280 nm using a UV detector, and molecular weights were calculated by comparing with the calibration curve.

### 2.9. Matrix-Assisted Laser Desorption/Ionization Time-of-Flight Mass Spectrometry (MALDI-TOF-MS)

The MALDI-TOF-MS of the digesta was conducted to more accurately determine the changes in the molecular weight of fibrils during gastric digestion [25]. Digested samples collected at different gastric digestion time points were adjusted to a concentration of 1 mg/mL and desalted using a 3000 Da filter membrane. Desalted samples were diluted with acetonitrile/water/trifluoroacetic acid solution and α-cyano-4-hydroxycinnamic acid (α-CHCA). Then, 2 μL of the diluted sample was dropped onto the sample stage of the mass spectrometer using a stainless-steel MALDI target plate with a standard ground-steel surface (Bruker MTP 384 target plate, Bruker Co., Billerica, MA, USA). Samples were analyzed using a MALDI-TOF-MS spectrometer equipped with a SmartBeam laser (Bruker Daltonics, Billerica, MA, USA). The measurement parameters were set as follows: positive linear mode, laser frequency 1000 Hz, acquisition range of 1000–10,000 Da, and acquisition range of 600–6000 Da in the positive reflector mode.

### 2.10. Fabrication of β-Carotene Nanoparticles Encapsulated by Native Protein/Fibrils

By referring to the research of [7], the amyloid fibril-β-carotene delivery system was prepared and the native protein group was regarded as a control. Firstly, β-carotene crystals were added to an appropriate amount of acetone to obtain a 0.25 mg/mL solution. Then, 6 mL of the β-carotene acetone solution was added to 15 mL of the above amyloid fibril or native protein solution (2 wt%), obtaining a β-carotene dispersion with a theoretical loading of 0.5 wt%. The above dispersion was stirred for 1 h in a 40 °C water bath, and during this period, the sample was subjected to ultrasonic treatment (200 W for 10 min, both working and break time are 2 s) to obtain a uniformly dispersed composite solution. The ultrasonicated solution was then subjected to a two-stage vacuum rotary evaporation. Under the 45 °C water bath, the sample was firstly evaporated at 150 mbar for 7 min and then at 90 mbar for 10 min to avoid foaming and overflow during rotary evaporation. After vacuum rotary evaporation, the β-carotene nanoparticles encapsulated by amyloid fibrils were obtained, which were sealed in a 50 mL plastic centrifuge tube and stored in a 4 °C refrigerator for further analysis. For ease of reading, the β-carotene delivery systems encapsulated by native protein and fibril were labeled as SP/BC and SPF/BC, respectively.

#### Physical Properties of β-Carotene Delivery System

The average diameter, ζ-potential, encapsulation efficiency and loading capacity of β-carotene delivery system encapsulated by native protein and amyloid fibrils were determined. The particle size of β-carotene nano-dispersions (diluted to 0.5 mg/mL with deionized water to minimize multiple scattering effects) was analyzed using a Zeta-sizer Nano (ZS90, Malvern, UK). The particle sizes were calculated using the Stokes-Einstein model and presented as accumulated mean diameters. The ζ-potential of samples was assessed using the Smoluchowski model based on the evaluation of electrophoretic mobility. The ζ-potential measurements were performed using disposable folded capillary cells (DTS1070, Malvern) filled with approximately 800 μL of the samples, and the electrophoretic mobility was recorded under the instrument’s automatically adjusted electric field (typically corresponding to 20–30 V/cm). All measurements were conducted in triplicate at 25 °C using the appropriate sample tanks.

The encapsulation efficiency and loading capacity of β-carotene-loaded delivery systems were calculated according to Zhang et al. [26]. β-Carotene was extracted from the nano-dispersion (1 mg/mL) using a mixture of absolute ethyl alcohol and n-hexane (1:3, *v*/*v*). The absorbance of the extract was measured at 450 nm using a UV-1800 spectrophotometer (Shimadzu Co., Kyoto, Japan), and β-carotene concentration was determined from a pre-established standard curve (y = 0.2623x + 0.0247, R^2^ = 0.9996). Encapsulation efficiency and loading capacity were then calculated using the following equations:

$$\mathrm{Encapsulation}\;\mathrm{efficiency}\;(\%)=\frac{\mathrm{The}\;\mathrm{mass}\;\mathrm{of}\;\mathrm{encapsulated}\;\beta -\mathrm{carotene}\;}{\mathrm{Total}\;\mathrm{mass}\;\mathrm{of}\;\beta -\mathrm{carotene}\;}\times 100\%$$

$$\mathrm{Loading}\;\mathrm{capacity}\;(\%)=\frac{\mathrm{The}\;\mathrm{mass}\;\mathrm{of}\;\mathrm{encapsulated}\;\beta -\mathrm{carotene}\;}{\mathrm{Total}\;\mathrm{mass}\;\mathrm{of}\;\mathrm{delivery}\;\mathrm{system}}\times 100\%$$

### 2.11. In Vitro Digestion of β-Carotene Nanoparticles Encapsulated by Native Protein/Fibrils

According to the method described by Lin et al. [27] with some modifications, a dynamic in vitro simulated digestive system (GI20, Nutriscan, Australia) was used to conduct the digestion experiments of β-carotene delivery system. The oral digestion was not involved because the delivery system was liquid in the absence of any starch-based materials.

*Gastric phase:* 20 mL of β-carotene dispersion solution with a pH of 2.5 was placed in a sample bottle and stored in a heating block, with the temperature maintained at 37 °C. 20 mL of simulated gastric digestive fluid (containing 2.0 mg/mL pepsin and 10 mM NaCl) was injected into the β-carotene dispersion solution at a rate of 2 mL/min through a peristaltic pump, while the stirring speed was set at 200 rpm. The digestion products were collected and used for further analysis at 60 min and 120 min after digestion. After the gastric phase digestion, the resulting mixture was adjusted to a pH of 7.0 using a pre-configured sodium hydroxide solution (1.0 M) to inactivate the digestive enzymes.

*Small intestinal phase*: The mixed digestion liquid obtained after the gastric phase (20 mL) was placed in a sample bottle and stored in a heating block, with the temperature maintained at 37 °C. Then, 20 mL of simulated intestinal fluid (SIF, containing 12.0 mg/mL bile salts, 2.0 mg/mL trypsin, 6.8 mg/mL KH_2_PO_4_ and 8.8 mg/mL NaCl) was injected into the gastric digestion liquid at a rate of 2 mL/min through a peristaltic pump, while the stirring speed was set at 200 rpm and the pH of the digestive fluid was adjusted to 7.0 in real time. The mixed digestion liquid was collected and used for further analysis at 60 min and 120 min after digestion.

After the small intestinal digestion, the bioaccessibility of β-carotene was determined. The small intestinal digestion fluid was transferred to a 50 mL centrifuge tube, centrifuged at 18,000× *g* for 60 min at 10 °C. The supernatant obtained from the centrifugation was filtered through a 0.45 μm filter membrane and defined as the mixed micellar phase. The bioaccessibility of β-carotene was determined by the following formula:

$$\mathrm{Bioaccessibility}\;\mathrm{of}\;\beta -\mathrm{carotene}\;(\%)=\frac{C_{micelle}}{C_{\mathrm{initial}\;\mathrm{dispersion}}}\times 100\%$$

where C_micelle_ and C_initial suspension_ represent the content of β-carotene in mixed micellar phase and the entire content of β-carotene encapsulated by native protein or fibrils, respectively.

### 2.12. Digestion Rates of Transport Carriers (Native Protein and Amyloid Fibrils)

The digestion rates of amyloid fibrils and native protein were determined using fluorescence-based assays according to Zhang et al. [13] and Wen et al. [28]. For amyloid fibrils, 20 mg/mL fibril samples were subjected to the simulated gastrointestinal digestion described above. After digestion, the samples were immediately placed in an ice bath to halt enzymatic activity before measurement. ThT fluorescence was measured to monitor fibril degradation, as ThT specifically bound to β-sheet structures in amyloid fibrils. The fibril digestion rate was calculated using the following equation:

Digestion rate of fibrils (%) = (I_0_ − I_1_)/I_0_ × 100%

where I_0_ and I_1_ indicate the ThT fluorescence intensities before digestion and at the particular digestion duration, respectively.

For native protein, the degree of hydrolysis was assessed by quantifying free amino groups released using the o-phthaldialdehyde (OPA) assay. Digested samples were centrifuged at 9000× *g* for 5 min at 4 °C, and the supernatant was collected. The supernatant was diluted 20-fold with 100 mM sodium carbonate solution and mixed 1:1 (*v*/*v*) with OPA reagent (100 mM tetraammonium bromide, 1 mg/mL SDS, 0.8 mg/mL p-naphthol, and 0.05 mg/L DTT) at room temperature for 10 min. The supernatant was diluted 20 times in order to reduce the sample concentration so that it fell within the linear detection range of the OPA assay. The 100 mM sodium carbonate provided an alkaline environment (pH 9–10), which was necessary for the OPA reagent to react efficiently with free amino groups. The fluorescence intensity of tryptophan residues was then measured at an excitation wavelength of 340 nm and an emission wavelength of 425 nm. These wavelengths were chosen because the OPA reagent reacted with free amino groups to form a fluorescent adduct whose emission was maximized at 425 nm upon excitation at 340 nm. A standard curve was established using tryptophan solutions at various concentrations (0.001–0.060 mM). The protein digestion rate was calculated as

Digestion rate of native protein (%) = h_s_/h_total_ × 100%

where h_s_ denotes the content of free amino groups produced after each gram of protein is digested (in mM), and h_total_ corresponds to complete hydrolysis.

### 2.13. Statistical Analysis

All measurements were performed in triplicate. Data were plotted using Origin software (Version 9.1, OriginLab, Northampton, MA, USA). The one-way Analysis of variance (one-way ANOVA) was conducted using SPSS software (IBM SPSS Statistics 19, Armonk, NY, USA) with a significance level of *p* < 0.05.

## 3. Results and Discussion

### 3.1. Changes in Normalized ThT Fluorescence Intensity and Apparent Viscosity of Amyloid Fibrils During Gastric Digestion

**Figure 1:**
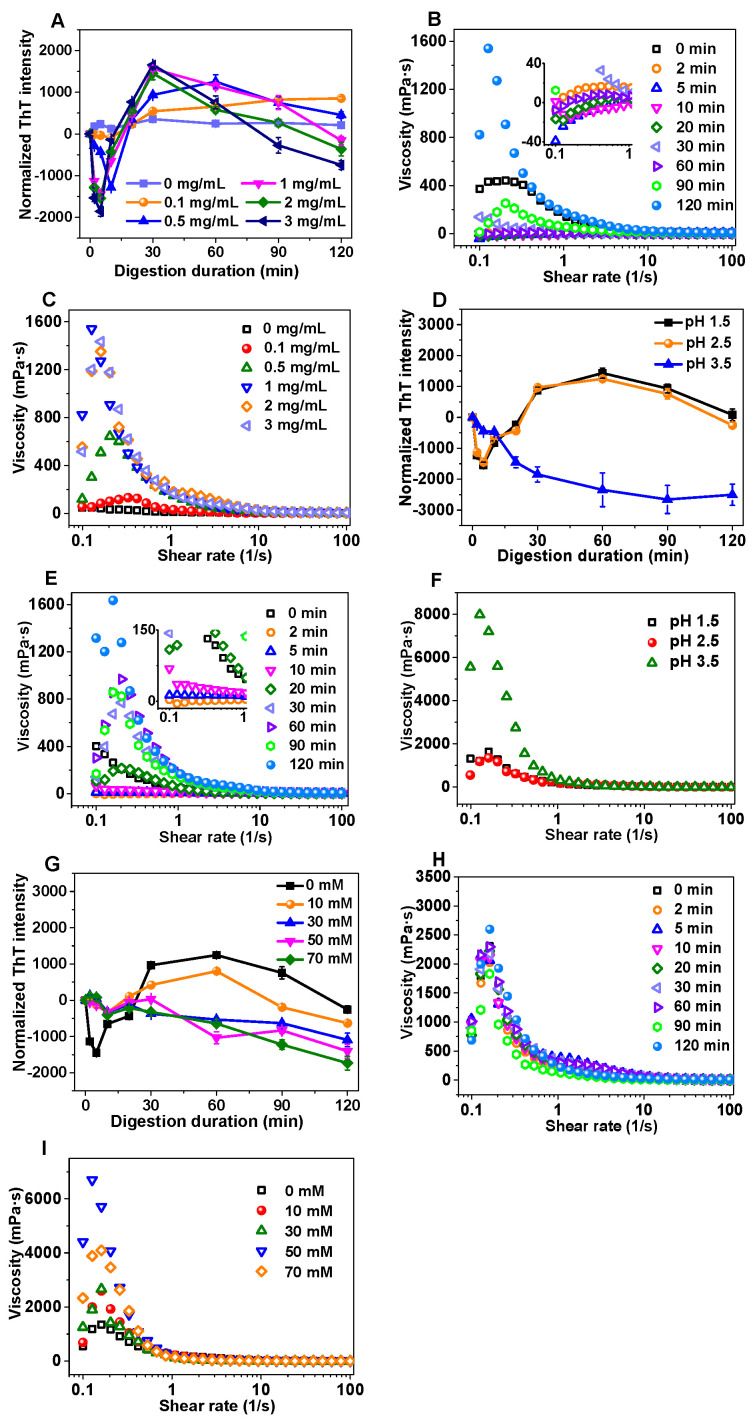
The effects of different concentrations of pepsin (0–3 mg/mL, (**A**–**C**), gastric digestion pH (1.5–3.5, (**D**–**F**)) and salt ion concentration (0–70 mM, (**G**–**I**)) on the normalized ThT fluorescence intensity and apparent viscosity of amyloid fibrils during gastric digestion. The approximate concentration of fibrils during the gastric digestion was 10 mg/mL.

To elucidate the kinetic changes in amyloid fibrils during gastric digestion, the effects of key gastric digestion parameters (pepsin concentration, pH and ionic strength) on normalized ThT fluorescence intensity (i.e., subtracting the ThT fluorescence intensity of the undigested sample) and apparent viscosity were investigated to reflect the enzymatic hydrolysis and potential regeneration of amyloid fibrils. As shown in Figure 1A, under different pepsin concentrations (0, 0.1, 0.5, 1, 2, and 3 mg/mL), the normalized ThT fluorescence intensity decreased initially, then increased, and finally decreased again as gastric digestion progressed. This trend became more pronounced with increasing pepsin concentration. During the early stage of gastric digestion (0–20 min), the normalized ThT fluorescence intensity of amyloid fibrils was negative, indicating that enzymatic hydrolysis dominated during this period, accompanied by a decrease in the apparent viscosity of the digest (e.g., pepsin concentration of 2 mg/mL, Figure 1B). The negative normalized fluorescence intensities occurred when early pepsin hydrolysis temporarily reduced ThT binding below the level of the intact fibrils. During the middle stage of gastric digestion (20–40 min), both normalized ThT fluorescence intensity and apparent viscosity gradually increased, suggesting that restructuring and regeneration of amyloid fibrils occurred during this period. The fluorescence intensity reached its maximum around 40 min, indicating a high regeneration rate of amyloid fibrils. This result was attributed to the rapid enzymatic hydrolysis during the early stage (0–20 min) with the accumulation of small peptide fragments, thus providing a favorable condition for fibril regeneration [23]. During the late stage of gastric digestion (40–120 min), the normalized ThT fluorescence intensity gradually decreased due to the reduced availability of small peptide fragments, thereby resulting in the insufficiency to support fibril regeneration. Additionally, the apparent viscosity of the digest increased to its maximum at 120 min during the late stage, possibly owing to the increased fibril length or aggregation (further confirmed via TEM observation of fibril morphology). As shown in Figure 1C, the apparent viscosity of the digest after 120 min of gastric digestion varied with pepsin concentration. The highest viscosity was observed at 1 mg/mL pepsin concentration, as excessively low pepsin concentration (e.g., 0.1 mg/mL) failed to initiate fibril regeneration, while excessively high concentration (e.g., 3 mg/mL) further hydrolyzed the fibrils and disrupted their original structure during the late stage (40–120 min) [9]. Therefore, based on the normalized ThT fluorescence intensity and apparent viscosity, a pepsin concentration of 2 mg/mL was selected for subsequent experiments of pH and ionic strength investigations.

Gastric pH undergoes slight variations due to factors such as food intake stimulation, gastric emptying and food buffering dilution. Thus, the impact of different gastric pH values (1.5, 2.5 and 3.5) on the gastric digestion characteristics of amyloid fibrils was investigated. As exhibited in Figure 1D, the normalized ThT fluorescence intensity of amyloid fibrils under pH 1.5 and 2.5 showed the similar trends, which was consistent with the changes aforementioned. This phenomenon suggested that the pepsin activity under pH 1.5 and 2.5 was comparable, exerting similar enzymatic effects on amyloid fibrils. Accordingly, the apparent viscosity of the digest at pH 2.5 showed an initial decrease followed by an increase, with the minimum and maximum viscosity observed at 2 min and 120 min of gastric digestion, respectively (Figure 1E). Notably, at pH 3.5, the normalized ThT fluorescence intensity gradually decreased, indicating slow enzymatic hydrolysis of amyloid fibrils without significant fibril regeneration. This might be because pH 3.5 was close to the isoelectric point of sesame protein (between 3.8 and 4.0), leading to reduced fibril solubility, aggregation and sedimentation, thereby preventing enzymatic hydrolysis and fibril regeneration [29]. This hypothesis was further supported by the significantly higher apparent viscosity of the digest at pH 3.5 as compared with that at pH 1.5 and 2.5 (Figure 1F). Therefore, pH 2.5 was selected as the representative condition for subsequent experiments on ionic strength.

Salt ions such as sodium, potassium and calcium (approximately 5–15 mM) are inevitably encountered during gastric digestion. In this study, sodium ions were selected as the representative salt ions to investigate the impact of different ionic strengths (0, 10, 30, 50 and 70 mM) on the gastric digestion behavior of amyloid fibrils. As shown in Figure 1G, at an ionic strength of 10 mM, the fluorescence intensity changes in the digest were similar to those of the control group. However, as the ionic concentration further increased, the normalized ThT fluorescence intensity gradually decreased, which indicated that the regeneration of amyloid fibrils was inhibited by sodium ions. This result might be because the dissociated Na^+^ and Cl^−^ competed with sesame protein fibrils for water molecules, weakening the critical hydration layer structure that maintained the stability of fibril dispersion [30]. Additionally, salt ions neutralized the surface charge of fibrils, reducing intermolecular electrostatic repulsion and promoting the fibril aggregation [31]. This explanation was confirmed by the higher apparent viscosity of the digest at ionic strength of 30 mM in Figure 1H,I. Therefore, in order to simulate the real gastric digestion environment (5–15 mM), a high level of salt ions was not added in the subsequent digestion experiments.

In summary, based on ThT fluorescence intensity changes and rheological properties, the digestion process of amyloid fibrils during gastric digestion was preliminarily characterized. Amyloid fibrils underwent enzymatic hydrolysis, reorganization and regeneration under the action of pepsin and gastric acid. Among them, enzymatic hydrolysis and reorganization had a direct influence on the regeneration pattern of amyloid fibrils. During this procedure, the pepsin concentration, pH and salt ion level all involved in the regulation of fibril regeneration. Based on the real gastric digestion environment and the measurements above, the optimal gastric digestion conditions for subsequent experiments were determined as pepsin concentration of 2 mg/mL, digestion pH of 2.5 and ionic strength of 10 mM.

### 3.2. SDS-PAGE Images of Amyloid Fibrils During Gastric Digestion

**Figure 2:**
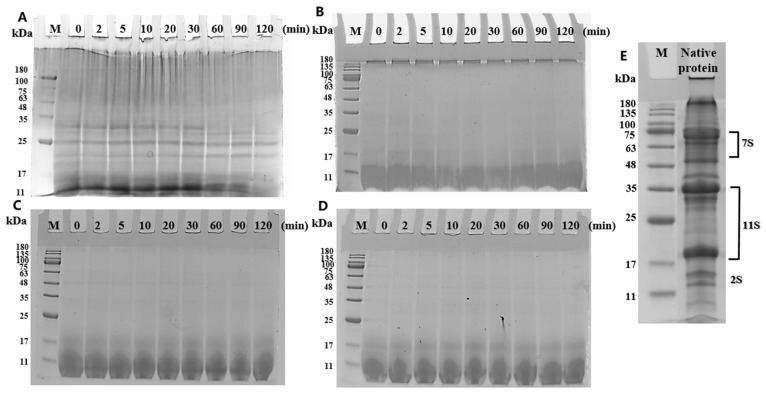
SDS-PAGE images of native sesame protein and amyloid fibrils under different gastric digestion conditions ((**A**): native protein, pH 2.5, 2 mg/mL pepsin + 10 mM NaCl; (**B**): fibrils, pH 2.5, without pepsin and NaCl; (**C**): fibrils, pH 2.5, 2 mg/mL pepsin; (**D**): fibrils, pH 2.5, 2 mg/mL pepsin + 10 mM NaCl; (**E**): native protein without any digestive components).

The molecular weight changes in native protein and amyloid fibrils during gastric digestion were displayed in Figure 2. For native sesame protein (Figure 2A), as compared with its initial image, the protein structure was rapidly hydrolyzed by pepsin within a short time, manifested as the rapid hydrolysis of the 7S protein component into peptides with molecular weights below 11 kDa, and partial hydrolysis of the 11S component. After 120 min of digestion, only several components with molecular weights of 22–25 kDa remained, suggesting that this component has high resistance to pepsin, while the rest was hydrolyzed into smaller peptides. This phenomenon indicated that native sesame protein was highly sensitive to pepsin and easily hydrolyzed into polypeptides and small peptides [32].

According to the study of [33], the formation mechanism of amyloid fibrils involves the hydrolysis of protein monomer peptide bonds, with the self-assembling of fibrillogenic amyloid peptide fragments into fibrillar aggregates. The non-migrating material at the top of SDS-PAGE lanes around 180 kDa (Figure 2B in the absence of pepsin) corresponded to unconverted native protein. As seen in the SDS-PAGE image (see Supplementary Materials), with the progression of fibrillization, native protein was hydrolyzed into polypeptides with the molecular weight lower than 17 kDa. However, the band of 180 kDa was still retained owing to the presence of unhydrolyzed proteins. By contrast, the bands of amyloid fibrils under different gastric digestion conditions did not change significantly, with the presence of bands below 11 kDa. This result illustrated that in the absence of pepsin, the molecular weight of amyloid fibrils remained unchanged, demonstrating their good resistance to gastric acid [34]. To address the concerns about SDS-mediated disruption of fibrils, the fibril morphology after sample-buffer treatment was examined by TEM in the Supplementary Materials. The fibril samples treated with SDS alone at room temperature retained the partial fibrillar morphology, whereas samples treated with SDS and DTT showed loss of fibrillar structure, confirming that the denaturing conditions used for SDS-PAGE converted fibrils into their constituent fragments.

After adding 2 mg/mL pepsin and 10 mM NaCl to the digest, no obvious bands were observed at the top of the lanes (Figure 2C,D), manifesting that large aggregates in amyloid fibrils were rapidly hydrolyzed upon contacting with pepsin. Notably, smaller bands (<17 kDa) remained unhydrolyzed throughout the gastric digestion, suggesting that amyloid fibrils possessed good tolerance to gastric digestion and could resist the destructive effects from gastric acid and pepsin. However, since SDS-PAGE could only provide a rough estimate of the molecular weight distribution of fibrils, the accurate molecular weight change during gastric digestion was unaccessible. Therefore, to further investigate the molecular weight variation in amyloid fibrils, techniques such as gel permeation chromatography (GPC) and matrix-assisted laser desorption/ionization time-of-flight mass spectrometry (MALDI-TOF-MS) were used to measure changes in the digesta in small molecular weights below 1000 Da, hence providing a theoretical basis for exploring the mechanism of amyloid fibril regeneration during gastric digestion.

### 3.3. Gel Permeation Chromatography of Amyloid Fibrils Before and After Gastric Digestion

**Figure 3:**
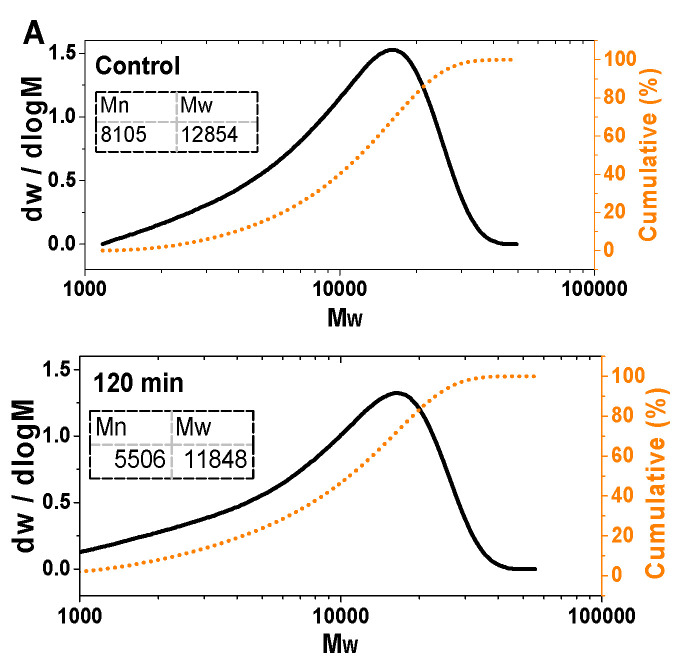

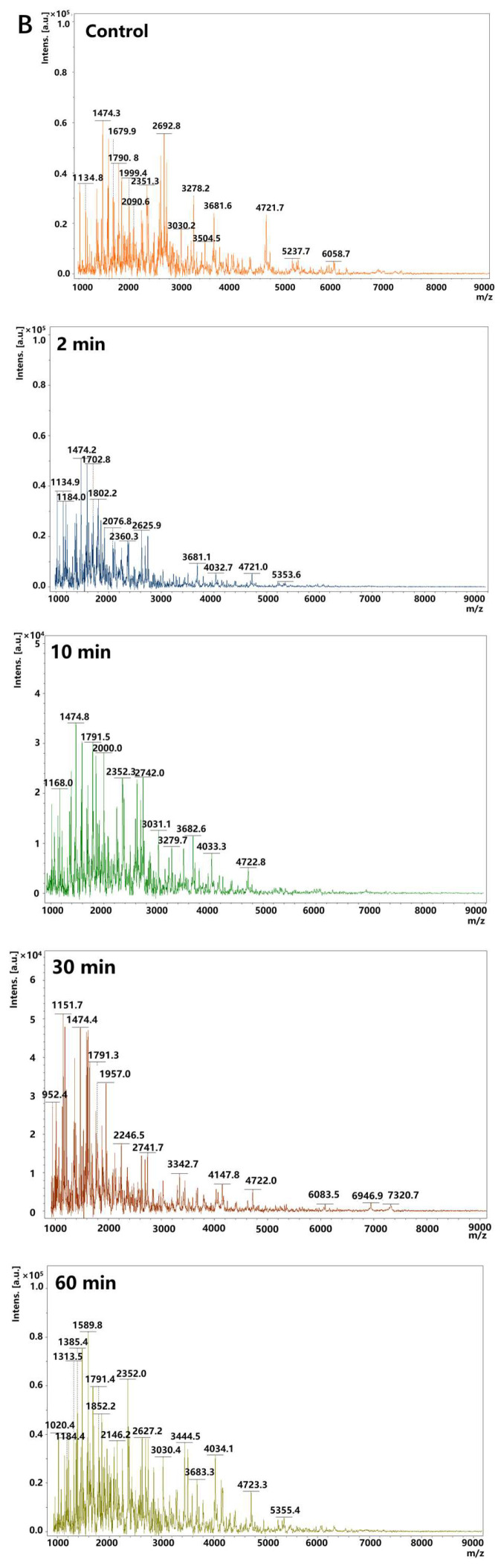

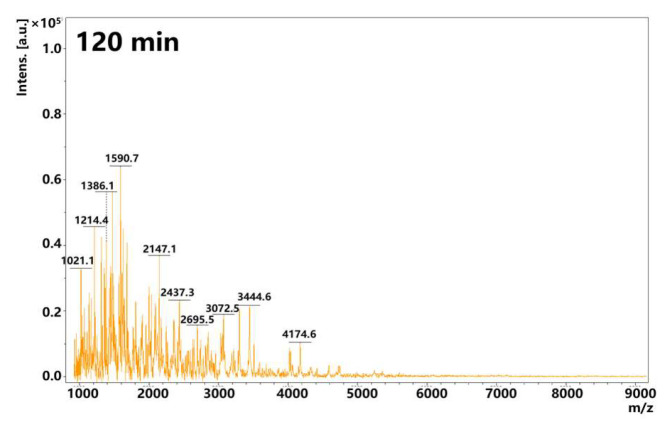
GPC (**A**) and MALDI-TOF-MS images (**B**) of sesame protein amyloid fibrils during gastric digestion.

The molecular weights of amyloid fibrils before and after gastric digestion were determined using GPC, as illustrated in Figure 3A. Before gastric digestion, undigested amyloid fibrils exhibited a broad molecular weight distribution, with the number-average (M_n_) and weight-average (Mw) molecular weights of 8105 and 12,854, respectively. These values were consistent with the SDS-PAGE results, indicating that the molecular weight of amyloid fibrils before digestion was concentrated around 11 kDa (native proteins were hydrolyzed into small molecular peptides during fibrillization). After gastric digestion, due to enzymatic degradation, the molecular weight distribution of amyloid fibrils narrowed. The M_n_ decreased from 8105 to 5506, implying the significant degradation of amyloid fibrils by pepsin. The degradation of amyloid fibrils led to the formation of low molecular weight fragments or the breaking of high molecular weight polymer chains into smaller units [10]. Meanwhile, the Mw decreased from 12,854 to 11,848 with a relatively small reduction extent, suggesting that high molecular weight components were partially degraded but still retained a proportion of large molecular structures. Additionally, the molecular weight was concentrated around 11 kDa (corresponding to SDS-PAGE bands) before digestion, while after digestion, Mw remained above 11 kDa and M_n_ significantly decreased, which was the symbol that the more fragments with low molecular weight were included in the enzymatic hydrolyzed products, while a small amount of undegraded component with high molecular weight was also retained [24]. This result might be associated with the regeneration of amyloid fibrils during the gastric digestion. In summary, gastric digestion reduced the molecular weight of amyloid fibrils through enzymatic action and narrowed their molecular distribution, reflecting the structural disruption of amyloid fibrils by enzymatic hydrolysis [35].

### 3.4. Mass Spectrometric Analysis of Amyloid Fibrils During Gastric Digestion

Due to the extremely high sensitivity and adaptability, mass spectrum is often used for qualitative and quantitative analyses of proteins and peptides [36]. MALDI-TOF-MS was used to further determine the molecular weights of digestion products of amyloid fibrils (Figure 3B) without the denatured treatment of fibrils. The detection in the low molecular weight range (1000–10,000 Da) would provide more detailed information on digestion products with low molecular weight. For control group (undigested amyloid fibrils), the mass spectrum mainly consisted of three major ion peaks below 3000 Da (1474.3, 1679.9 and 2692.8 Da), along with three minor ion peaks above 3000 Da (3278.2, 3681.6 and 4721.7 Da). This result indicated that amyloid fibrils were composed of heterogeneous peptide components with a polymerization pattern possibly involving the synergetic assembly of a stable core (small molecular weight peaks) and peripheral easily degradable regions (large molecular weight peaks) [37]. After 2 min of gastric digestion, the peak intensities of the three minor ion peaks above 3000 Da rapidly decreased, and the intensity of the 2692.8 Da ion peak also significantly decreased. This status suggested that the high molecular weight regions might correspond to loose structures or surface-exposed regions of amyloid fibrils, which were more easily recognized and cleaved by pepsin. These components might form aggregates through non-covalent interactions (e.g., hydrophobic interactions or hydrogen bonds) and exhibit weak stability during gastric digestion [11]. As gastric digestion progressed, the intensities of ion peaks below 3000 Da gradually increased. For example, at 30 min of digestion, a major ion peak appeared at 1157.1 Da, indicating that pepsin continued to exert enzymatic effect during this period, producing smaller oligopeptide fragments. As digestion time further prolonged, the intensities of ion peaks above 3000 Da increased again, such as peaks at 3444.5, 4034.1 and 3072.5 Da reappeared. This appearance indicated that the low-molecular-weight oligopeptides (such as 1157.1 Da) generated by the continuous hydrolysis of pepsin might reaggregate through non-covalent interactions (hydrophobic interactions, hydrogen bonds) to form stable macromolecular skeleton [38]. For example, some enzymatic hydrolysis fragments were likely to be polymerized due to exposed hydrophobic sequences, forming amyloid fibrils with new β-sheet structures [39]. Thus, it was reasonable to infer that the digestion of amyloid fibrils in the stomach was a competitive process, where partially hydrolyzed products might act as seeds to promote the aggregation of other oligopeptide fragments, leading to the reappearance of large molecular weight peaks. This judgment was consistent with the theory of disaggregation, reorganization and regeneration of amyloid fibrils during gastric digestion aforementioned.

### 3.5. Morphological Changes in Amyloid Fibrils During Gastric Digestion

**Figure 4:**
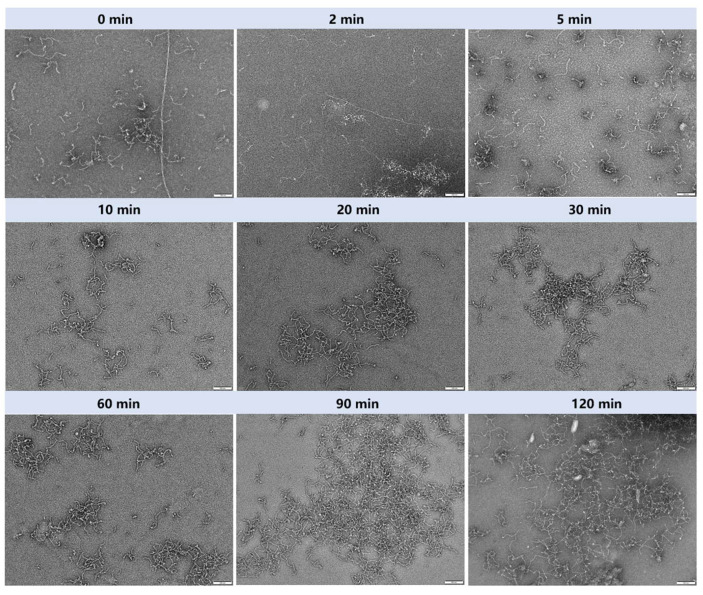

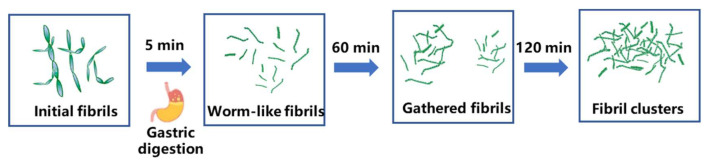
TEM images and evolution process of sesame protein amyloid fibrils during gastric digestion (scale bar 100 nm).

To clarify the state of amyloid fibrils after gastric digestion, TEM was used to observe and analyze the morphology of fibrils before and after gastric digestion and also to elucidate the property difference between regenerated and original fibrils. The TEM images were displayed in Figure 4. Before gastric digestion, amyloid fibrils consisted mostly of curved worm-like flexible fibrils, accompanied by a few long-rigid fibrils (length exceeding 1 μm). As gastric digestion progressed, the long-rigid fibrils gradually disappeared and were completely absent after 5 min of digestion, replaced by worm-like fibrils (approximately 300 nm in length). This result unraveled that rigid fibrils were highly sensitive to gastric digestion conditions (e.g., low pH, pepsin action), and their degradation might be related to the disaggregation of β-sheet structures or enzymatic cleavage. In contrast, worm-like flexible fibrils remained at approximately 300 nm in length after gastric digestion, suggesting that their conformation (e.g., curved morphology, internal hydrophobic interactions) resisted the destructive effect of the gastric environment [40]. As gastric digestion time further increased, amyloid fibril fragments began to aggregate into clustered structures (e.g., at 20, 30 and 60 min of digestion), followed by the aggregation of clustered structures into network-like structures (e.g., at 90 and 120 min of digestion). This phenomenon might be ascribed to that enzymatically hydrolyzed short fibrils expose more hydrophobic regions or charged groups, thus promoting hydrophobic interactions or electrostatic attraction between fragments and inducing clustered aggregation. In addition, under the low pH condition of the stomach, continuous stirring could also accelerate the regeneration of original fibrils [41]. In other words, the decomposition products of the β-sheet (such as short fibrils) might serve as nucleation sites, and under the action of mechanical stirring (simulating gastric peristalsis), the regeneration process of the original fibrils was triggered [10].

### 3.6. Physical Properties of β-Carotene Delivery System

**Figure 5:**
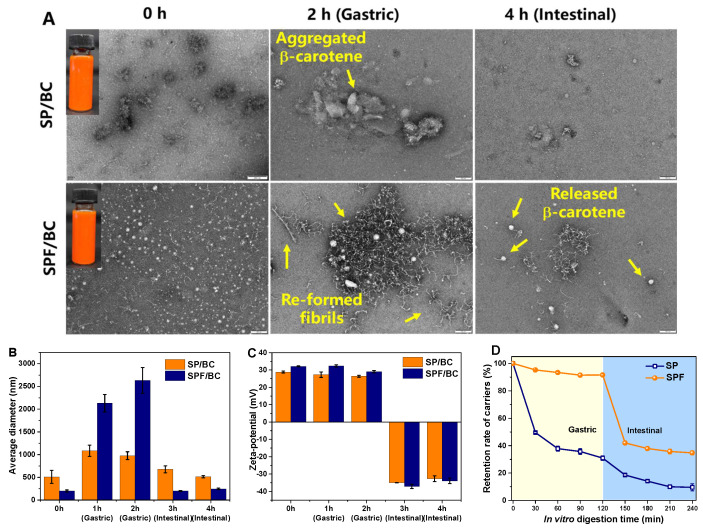

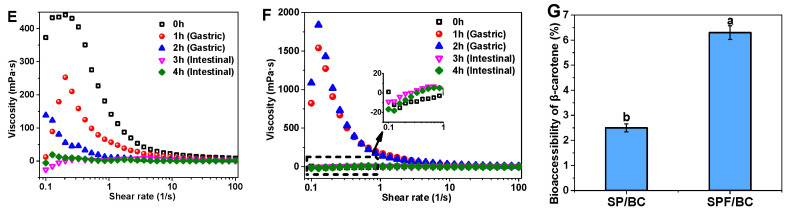
Microscopic structural changes in native protein and fibril-based delivery systems during the digestion process (scale bar 100 nm) (**A**), particle size (**B**), z-potential (**C**), carrier retention rate (**D**) and apparent viscosity ((**E**) denotes SP/BC and (**F**) denotes SPF/BC) of digestive juices during the digestion, bioaccessibility of β-carotene after the digestion ((**G**), different superscript letters (a and b) in the figure indicate significant differences (*p* < 0.05)).

Generally, as for delivery systems of nutrients, smaller particle size and uniform particle size distribution are closely related to its excellent physical stability and bioaccessibility. The TEM images, average diameter, ζ-potential, encapsulation efficiency and loading capacity values of β-carotene delivery systems were exhibited in Figure 5A and Table 1, respectively.

When using native protein as the transport carrier, the particle size of the obtained β-carotene dispersion was relatively large (about 500 nm), and obvious complex aggregation could be found in the TEM image, indicating that the hydrophobic characteristics of sesame protein and β-carotene might not be effectively matched during the encapsulation process. Since β-carotene was a highly lipophilic substance, if the hydrophobic groups of the carrier protein were not fully exposed, the lack of hydrophobic interaction would lead to insufficient binding between the two components, thereby causing aggregation [42]. When amyloid fibrils were employed as transport carriers, the particle size of the delivery system was reduced to around 200 nm, and the ζ-potential increased to a higher level. This result indicated that amyloid fibrils with a linear framework structure were more likely to form a uniform loading interface compared to natural spherical proteins. The lipophilic β-carotene molecules could be effectively anchored onto these fibrils through hydrophobic interactions and hydrogen bonds, reducing phase separation caused by insufficient exposure of hydrophobic groups [7]. In addition, the increase in the ζ-potential of the fibril indicated an increase in the surface charge density of the carrier, which could also inhibit the aggregation of β-carotene nanoparticles through electrostatic repulsions.

During storage and consumption, the transport system is required to have a high encapsulation efficiency and loading capacity to prevent the dose loss due to the first-pass effect during digestion. Compared with native protein transport carrier, the fibril carrier had better embedding properties, and the resulting transport system possessed a higher encapsulation efficiency and loading capacity of β-carotene (91.0% and 0.31%, respectively). This result was attributed to that the fibrillization procedure promoted the unfolding of native protein molecules, allowing the internal hydrophobic amino acid residues (such as phenylalanine, leucine) to be fully exposed, thereby facilitating the formation of a more stable complex between the fibril and the conjugated double bond system of β-carotene through hydrophobic interactions [16].

### 3.7. In Vitro Simulated Digestion of β-Carotene Transport Systems

The in vitro digestion behavior of hydrophobic active compounds is crucial for evaluating the potential applications of delivery carriers. As shown in Figure 5B, after gastric digestion, the particle size of all samples increased sharply. The increment in the particle size of SP/BC was due to the aggregation of β-carotene particles, and large β-carotene particles could be clearly seen in TEM image (Figure 5A). The increased particle size was not associated with the digestion of protein since native protein was more sensitive to pepsin. As displayed in Figure 5D, SP was rapidly enzymatically degraded into peptides in the early stage of digestion, hence barely having an impact on the particle size of digestion fluid. For fibril carriers, the increase in particle size was owing to the regeneration of the fibrils, where the original fibrils gathered with each other to form new fibrils with the clustered structure. The reformed-fibril carriers effectively encapsulated the β-carotene particles, forming a shell protective layer (Figure 5A). This structure could effectively prevent direct contact between the digestive fluid and β-carotene nanoparticles, improving the stability of the β-carotene in the gastric fluid [43]. After small intestinal digestion, all carriers were further enzymatically degraded by pancreatic proteases, where the fibrils were rapidly enzymatically degraded (Figure 5D). The particle size of digesta decreased rapidly with the ζ-potential values changing from positive to negative (Figure 5B,C), making it hard to observe the specific microscopic structure in the digestive fluid. From TEM images, it could be noticed that the aggregation of β-carotene particles could still be observed in SP/BC, while in SPF/BC, the released β-carotene nanoparticles could be observed, and no obvious aggregation was found.

The rheological properties of each digestive fluid during the gastrointestinal digestion also underwent significant changes. As shown in Figure 5E,F, the viscosity of the digestive fluid of SP/BC continuously decreased, which was related to the continuous enzymatic degradation of proteins. In Figure 5D, the native protein was continuously enzymatically degraded into small molecular peptides by gastric proteases and pancreatic enzymes at the gastric and small intestinal stages, thereby resulting in a significant decrease in the viscosity. In contrast, the digestive fluid of SPF/BC showed a significant increase in viscosity during the gastric digestion stage. This phenomenon was attributed to the structural regeneration behavior of amyloid fibrils in the acidic gastric fluid environment. The fibrils gathered with each other and re-organized under the low pH, forming a new fibril network. This structural reorganization induced aggregation between fibrils, resulting in an increase in the viscosity of the gastric digestive fluid.

Once the gastrointestinal digestion was completed, the final bioaccessibility of β-carotene was determined in Figure 5G. Due to the significant aggregation of β-carotene during gastric digestion in SP/BC, large β-carotene crystalline aggregates were formed, limiting the dissolution and release of β-carotene in the small intestine [44]. Therefore, the bioaccessibility of β-carotene in SP/BC was relatively low (2.52%). However, the bioaccessibility of SPF/BC was significantly (*p* < 0.05) improved, which was attributed to that the stable network structure formed by the regenerated fibrils effectively inhibited the excessive aggregation of β-carotene, forming a more uniform dispersion system and promoting the release of β-carotene in the small intestine. This result further indicated that sesame protein-derived fibrils could be used as an efficient functional delivery carrier in improving the gastric digestive stability of hydrophobic nutrients.

## 4. Conclusions

In this study, the gastric digestion of amyloid fibrils and their potential as a delivery system for β-carotene were thoroughly investigated. The results demonstrated that amyloid fibrils underwent enzymatic hydrolysis and reorganization during gastric digestion, leading to their partial degradation and regeneration. Key factors such as pepsin concentration, pH and ionic strength were found to significantly influence the digestion behavior, with pepsin concentrations of 2 mg/mL, pH 2.5 and ionic strength of 10 mM providing optimal conditions for fibril regeneration. TEM, SDS-PAGE, and mass spectrometry confirmed that amyloid fibrils exhibited good resistance to gastric digestion, maintaining their structure and supporting β-carotene encapsulation. When used as a carrier, amyloid fibrils reduced the particle size and enhanced the stability of β-carotene, preventing aggregation during gastric digestion. Moreover, the regenerated fibril structure effectively protected β-carotene from excessive aggregation, improving its bioaccessibility in the small intestine.

However, several limitations of this study should be acknowledged. Firstly, the digestion experiments were conducted under in vitro simulated gastrointestinal conditions, which could not fully recapitulate the highly dynamic physiological environment of the human gastrointestinal tract (including peristalsis, mucosal interactions and complex enzymatic regulation). Secondly, this study specifically examined regenerated fibrils derived from sesame protein and β-carotene as a model hydrophobic nutrient; therefore, the generalizability of the delivery performance to other proteins or bioactive compounds remains uncertain. Furthermore, the cytotoxicity, immunogenicity and in vivo metabolic consequences of the regenerated fibrils were not evaluated. Future studies should incorporate in vivo validation and bioactivity models to fully assess the applicability and biosafety of fibril-based delivery systems.

## Figures and Tables

**Table 1:** The average diameter, ζ-potential, encapsulation efficiency and loading capacity of β-carotene delivery system.

Samples	Average Diameter (nm)	ζ-Potential (mV)	Encapsulation Efficiency (%)	Loading Capacity (%)
SP/BC	510.3 ± 144.7 ^a^	28.8 ± 0.6 ^b^	84.2 ± 0.3 ^b^	0.17 ± 0.05 ^a^
SPF/BC	198.8 ± 23.7 ^b^	32.1 ± 0.2 ^b^	91.0 ± 0.2 ^a^	0.31 ± 0.04 ^a^

Values are means ± SD (n = 3). Different superscript letters in the same column indicate significant differences (*p* < 0.05).

## Data Availability

The original contributions presented in this study are included in the article/Supplementary Materials. Further inquiries can be directed to the corresponding authors.

## Supplementary Materials

The following supporting information can be downloaded at: https://www.mdpi.com/article/10.3390/nano15231829/s1, Figure S1. SDS-PAGE image of sesame protein at different durations (0, 0.5, 1, 2, 4, 6, 9, 12 and 24 h) during fibrillization. Figure S2. TEM images of untreated fibrils (A) and fibrils treated by 10 mg/mL SDS (B) and 10 mg/mL SDS and 50 mM DTT (C) (scale bar 100 nm).
