# Supplementary material for: The Reconstruction of Sesame Protein-Derived Amyloid Fibrils Alleviates the Gastric Digestion Instability of β-Carotene Nanoparticles
Source: Nanomaterials (Basel). 2025 Dec 3;15(23):1829. doi: 10.3390/nano15231829 (PMC12693544; doi:10.3390/nano15231829)
Supplement: Supplementary file 1 [file nanomaterials-15-01829-s001.zip › nanomaterials-3953149-supplementary.pdf]

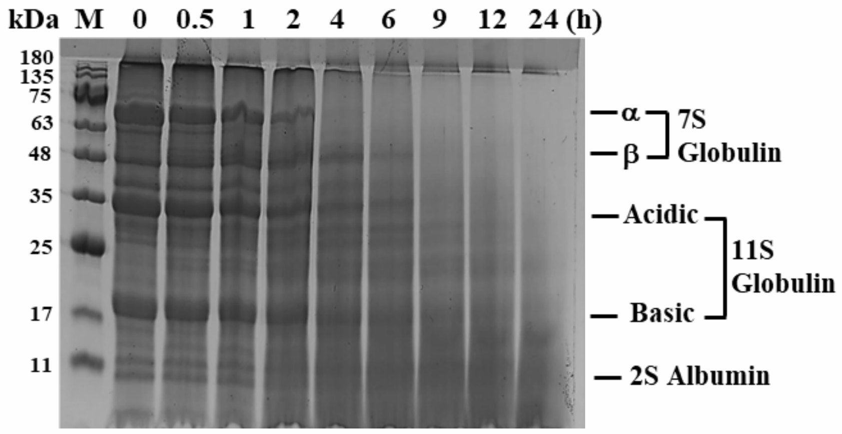

Figure S1. SDS-PAGE image of sesame protein at different durations (0, 0.5, 1, 2, 4, 6, 9, 12 and 24 h) during fibrillization.

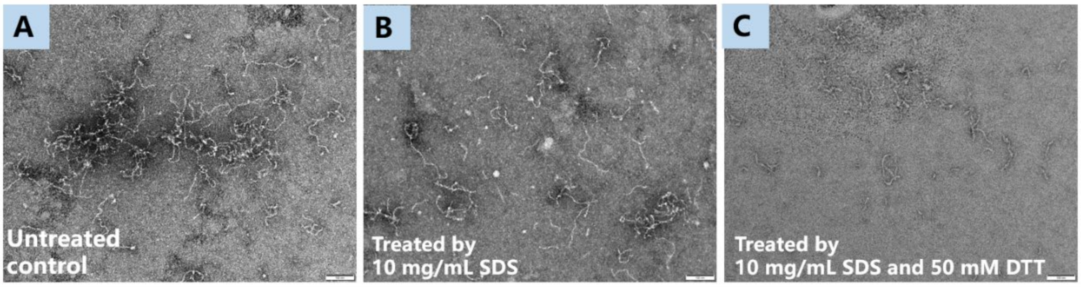

Figure S2. TEM images of untreated fibrils (A) and fibrils treated by 10 mg/mL SDS (B) and 10 mg/mL SDS and 50 mM DTT (C) (scale bar 100 nm).
